# The Book World of Medicine and Science

**Published:** 1905-11-11

**Authors:** 


### THE BOOK WORLD OF MEDICINE AND SCIENCE.

MATERIA MEDICA AND THERAPEUTICS. By J. MITCHELL BRUCE, M.A., M.D. Lond. (London: Cassell and Co., Ltd. 1905. Pp. 632. Price 7s. 6d.)

THE fact that, during a period of about twenty years, a book has passed through seven editions is sufficient guarantee of its utility. The seventh revised edition of Mitchell Bruce's compact Materia Medica has been brought up to the level of our latest knowledge. A new part has been added, comprising the materia medica and therapeutics of the drugs of the Indian and Colonial addendum to the British Pharmacopœia. This and other small additions have added forty-eight pages to the book. Mitchell Bruce's Materia Medica must continue to hold a prominent place in the library of every student and practitioner of medicine.

A TEXT-BOOK OF THE PRINCIPLES OF HYGIENE BASED ON PHYSIOLOGY. For the use of school teachers. By A. WATT SMYTH. (London: Simpkin, Marshall, Hamilton, Kent and Company, Limited. 1905. Pp. 256, 16 plates. 25 illustrations. Price 6s.)

WHILE the author has preferred to describe this as a text-book of hygiene the bulk of the work is more largely and more directly concerned with physiology, though he does not fail to draw practical deductions from his statements, or put the reader in a position to do so for himself. The importance of a practical knowledge of physiology to a teacher can scarcely be exaggerated, and in this volume may be found ample material to guide him in management of his pupils, both individually and collectively. While of necessity somewhat condensed the matter is well arranged and clearly put forward, so that though the book contains more than many of higher pretensions it is remarkably pleasant and easy to read. It is to be regretted, however, that more use has not been made of graphic methods of representation which so greatly aid in correctly grasping a subject and consequently assist the memory. This is of special importance where the absence of a preliminary training in anatomy leaves room for very vague impressions as to many important structures. Even photographic plates, and those of the author are worthy of commendation, do not compensate for the scarcity of diagrams in the text. Indeed, it may be said that a few good diagrams of the heart are more useful to the student of physiology than the excellent photographs here provided, while the skiagrams and dissection of the sole of the foot are almost superfluous. The book is one which deserves to be read by a wider public than that for which it is primarily designed and should be in the hands of all teachers. It is well printed, tastefully bound, and, most important of all, amply and carefully indexed.

